# *Citrus aurantium* increases seizure latency to PTZ induced seizures in zebrafish thru NMDA and mGluR's I and II

**DOI:** 10.3389/fphar.2014.00284

**Published:** 2015-02-13

**Authors:** Coral Rosa-Falero, Stephanie Torres-Rodríguez, Claudia Jordán, Rígel Licier, Yolimar Santiago, Zuleyma Toledo, Marely Santiago, Kiara Serrano, Jeffrey Sosa, José G. Ortiz

**Affiliations:** ^1^Neuropharmacology Laboratory, Pharmacology and Toxicology Department, University of Puerto Rico-Medical Sciences CampusSan Juan, PR, USA; ^2^School of Science and Technology, Universidad del EsteCarolina, PR, USA

**Keywords:** natural products, *Citrus aurantium*, zebrafish, epilepsy, glutamate, Puerto Rican folklore

## Abstract

Epilepsy is a serious neurological condition and pharmacotherapy is not effective for all patients and causes serious adverse effects and pharmacokinetic and pharmacodynamic interactions. Natural products and ethnobotanical resources can help develop new therapeutic options for conditions like epilepsy. In Puerto Rico, ethnobotanical resources highlight the anxiolytic properties of a tea like preparation made from the leaves of the *Citrus aurantium* tree or bitter orange. Studies performed with essential oils from the peel of the fruit have shown to increase seizure latency to pentylenetetrazole (PTZ) and maximal electroshock seizure in mice. We characterized the extract composition, and used a model of PTZ induces seizures in the zebrafish and a receptor-ligand binding assay to determine if this preparation has anticonvulsant properties and its mechanism of action. We determined that the aqueous extract made from the leaves of the *C. aurantium* tree contains hesperidin, neohesperidin, and neohesperidin dihydrochalcone. Using our zebrafish model, we determined that exposure to the *C. aurantium* 28 mg/mL extract in aquarium water increases seizure latency by 119% compared to controls. We ruled out a mechanism involving GABA_A_ receptors using the selective antagonist gabazine. We used two approaches to study the role of glutamate in the mechanism of the *C. aurantium* extract. The ligand binding assay revealed *C. aurantium* extracts at concentrations of 0.42 to 5.6 mg/mL significantly reduced [^3^H]Glu binding indicating an interaction with glutamate receptors, in particular with NMDA receptors and mGluR II. This interaction was confirmed with our animal model using selective receptor antagonists and we identified an interaction with mGluR I, not observed in the ligand binding experiment. These study provide evidence of the anticonvulsant properties of the aqueous extract made from the leaves of the *C. aurantium* tree and a mechanism involving NMDA and mGluR's I and II.

## Introduction

Epilepsy affects 50 million people worldwide (Meyer et al., 2010). It is a disorder of brain function characterized by the periodic and unpredictable occurrence of seizures caused by abnormal neuronal firing. It is a lifelong condition that requires continuous use of antiepileptic drugs (AED's) but the ones clinically available are not effective in all patients and, because of repeated use, patients develop tolerance to their medication (Löscher and Schmidt, 2006). In addition, AED's have serious adverse effects including sedation and drowsiness, cognitive impairment, hirsutism, weight gain (Cramer et al., 2010), and complex pharmacokinetic and pharmacodynamic interactions with an extensive number of commonly used medications (Patsalos and Perucca, 2003). These issues highlight the need for new and better therapeutic agents to replace current available options or to improve their effects. This can be achieved using natural products and ethnobotanical resources to identify possible candidates for drug development.

Ethnobotanical resources from Puerto Rico document the use of a tea made from the leaves of the *Citrus aurantium* tree as an anxiolytic remedy (Hernández et al., 1984; Alvarado-Guzmán et al., 2009) although there are is no scientific evidence documenting these properties. In experimental models of epilepsy, the essential oils from the peel of the fruit were able to increases seizure latency to pentylenetetrazole (PTZ) and maximal electroshock seizure in mice (Carvalho-Freitas and Costa, 2002). These essential oils also reduce anxiety like behavior in mice (Pultrini et al., 2006). Since the essential oils of the peel have anticonvulsant properties, it is important to determine if the aqueous extract made from the leaves of the *C. aurantium* tree also possess anticonvulsant properties.

Natural products are a valuable source of possible therapeutic agents since they tend to have fewer side effects than conventional therapeutic agents (Reeta et al., 2011). Also, extracts from natural products can be refined and modified to take advantage of their properties and develop therapeutic options. Identifying natural products with anticonvulsant properties could lead—in the future—to the development of new therapeutic options (Dias et al., 2012; Lahlou, 2013). Using a model of PTZ induced seizures in zebrafish, we documented the anticonvulsant properties of this aqueous extract and established a possible mechanism involving NMDA and mGluR's I and II. To our knowledge, this is the first time and aqueous extract made from the leaves of the *C. aurantium* tree has been studied, giving insight to the pharmacological properties of a preparation commonly used by patients, particularly in Puerto Rico. This work supports the value of ethnobotanical resources and folk medicine during the identification of new therapeutic compounds.

## Materials and methods

### Animal husbandry

Adult wild type zebrafish (*Danio rerio*), 3–6 month-old male and female and approximately 0.25 ± 0.04 g in weight were obtained from a local commercial distributor (Caribe Fisheries, Inc., Lajas, PR). Animals were housed in acrylic tanks covered with blue contact paper to reduce stress caused by traffic in the laboratory room dedicated as satellite facility for housing. Animals are kept at a density of approximately 5 animals per liter of water. These tanks were maintained with deionized tap water, supplemented with 60 mg/L Instant Ocean® Sea Salt (Spectrum) to achieve the appropriate water chemistry (Reed and Jennings, 2011). Fish tanks were maintained with constant filtration systems (mechanical and biological). The water temperatures were maintained at 25–27°C using water heaters. Illumination was provided by ceiling-mounted fluorescent light tubes on a 14 h light/10 h dark cycle. Fish were allowed a week in quarantine to acclimate to their new environment and determine health condition before any experiment was performed. Animals were hand fed at least once a day and fed by an automatic dispenser twice a day with Tetramin® tropical fish flake food (Tetra Co.) or Wardley tropical fish flake food depending on availability. All experimental manipulations were performed between 8 a.m. and 5 p.m. in a designated bench on the main laboratory room.

These experiments were performed in accordance with the recommendations in the Guide for the Care and Use of Laboratory Animals of the National Research Council (US) Committee for the Update of the Guide for the Care and Use of Laboratory Animals to minimize pain and distress to the animals. The Protocol was approved by the Institutional Animal Care and Use Committee of the University of Puerto Rico, Medical Sciences Campus (Protocol 3180110). An *n* = 10–15 animals per variable was selected as this range proves enough to establish statistically valid difference between control and experimental groups (Wong et al., 2010). Animals showing signs of distress either in the housing tanks or during experiments were humanely euthanized following institutional IACUC regulations.

### Chemicals

L-[2,3,4-^3^H]-Glutamic acid ([^3^H]Glu) (60 Ci/mmol) and was obtained from American Radiolabeled Chemicals, Inc. (St. Louis, MO). N-Methyl-D-aspartic acid (NMDA, 99% purity, 105 mM in Tris-HCl buffer pH 7.4), kainic acid (KA, 98% purity, 10 mM stock in Tris-HCl buffer pH 7.4), Fluorowiillardiine (FW, 98% purity, 10 mM stock in Tris-HCl buffer pH 7.4), (L)-(+)-α-Amino-3,5-dioxo-1,2,4-oxadiazolidine-2-propanoic acid (QA, 99% purity, 10 mM stock in Tris-HCl buffer pH 7.4), (2S,1'S,2'S)-2-(Carboxycyclopropyl)glycine (LCCG-I, 99% purity, 100 mM stock in in Tris-HCl buffer pH 7.4), (DCG-IV, 98% purity, 100 mM stock in Tris-HCl buffer pH 7.4), L-(+)-2-Amino-4-phosphonobutyric acid (L-AP4, 99% purity, 100 mM stock in Tris-HCl buffer pH 7.4), N-Phenyl-7-(hydroxyimino) cyclopropa[b]chromen-1a-carboxamide (PHCCC, 98% purity, 4 mM in 40% Tris buffer/60% DMSO) (2S)-a-Ethylglutamic acid (EGLU, 95% purity, 100 mM in 1 M NaOH), (RS)-α-Cyclopropyl-4-phosphonophenylglycine (CPPG, 98% purity, 25 mM stock in DMSO), 2,3-Dioxo-6-nitro-1,2,3,4-tetrahydrobenzo[f] quinoxaline-7-sulfonamide disodium salt (NBQX, 98% purity, 100 mM stock in dH_2_O), D-(-)-2-Amino-5-phosphonopentanoic acid (D-AP5, 99% purity, 100 mM stock in dH_2_O), (αS)-α-Amino-3-[(4-carboxyphenyl)methyl]-3,4-dihydro-5-iodo-2,4-dioxo-1(2H)-pyrimidinepropanoic acid (UBP 301, 98% purity 5 mM stock in DMSO), 2-(3-Carboxypropyl)-3-amino-6-(4 methoxyphenyl)pyridazinium bromide (gabazine, 98% purity, 10 mM in dH_2_O) were obtained from Tocris Bioscience or Abcam. Potassium Chloride was purchased from Matheson Coleman & Bell (Norwood, OH). UniverSol ES was obtained from MP Biomedicals (Solon, OH). Pentylenetetrazole (PTZ, 99% purity, 4 M stock in dH_2_O) and all other reagents were obtained from Sigma-Aldrich.

### Preparation of the *C. aurantium* extract

*C. aurantium* leaves were obtained from local trees in the Eastern and Northern Region of the island of Puerto Rico. The leaves were gently cleaned using a moist paper towel to remove dust and dirt prior extract preparation and heated in ultrapure water at 32°C for 1 h to obtain an aqueous extract that was filtered through a 12.5 cm Whatman Qualitative no. 1 filter. Supplementary Figure 3 shows a representative photo of the leaves used. A voucher specimen was deposited at the herbarium of the Botanical Garden of the University of Puerto Rico. Voucher number is pending to be assigned.

### Chemical analysis of the *C. aurantium* extract

Chemical characterization of the *C. aurantium* extract was performed by ChromaDex Inc. Briefly, the extract was prepared as described above and stored at −80°C for approximately 5 h and shipped overnight to the ChromaDex facilities. According to the results report, for all the analysis, the sample was prepared by filtering neat through a 0.45 μM PTFE filter into a gas chromatography (GC) or high performance liquid chromatography (HPLC) vial for analysis. For the limonene analysis, separation was achieved using a HP7890 GC Series II Plus with an FID detector. The sample was eluted with helium gas and separated using a 2B-50 column (30.0 m × 0.25 mm × 0.25 μm) and an injection volume of 1 μL. For the flavonoid analysis, separation was achieved using a Agilent 110 series HPLC system with UV-Vis detection with a Phenomenex Luna C18(2) 250 × 4.6 mm, 5 μm column operated at 40°C, a gradient elution with two mobile phases, mobile phase A: 10 mM Ammonium Acetate pH 5.0, mobile phase B: 9:1 Acetonitrile(ACN):Methanol(MeOH), a flow rate of 1.5 mL/min and an injection volume of 10 μL. For the synephrine alkaloids and related amines, besides the direct sample, a 10× dilution was also prepared by diluting the neat with half volume 0.1% phosphoric acid in water and half volume 20 mM borate buffer and filtered as described into the sample vials. Separation was achieved using a Agilent 1100 Series HPLC system with UV-Vis detection, a Phenomenex Luna C18(2) 250 × 4.6 mm, 5 μm column operated at 35°C, a gradient elution with two mobile phases, mobile phase A: 10 mM HAS in borate buffer and mobile phase B in 20:80//CAN-Borate and 10 mM HSA, a flow rate of 0.850 mL/min and injection volume of 20 μL.

### Cerebral cortex synaptic membranes

Synaptic membranes were prepared by Analytical Biological Services, Inc. (Wilmington, DE) as follows: female rats of approximately 2 months of age were decapitated and the brain promptly removed. The cortex was dissected and homogenized (1:10 w/v) in ice-cold 10 mM Tris-HCl buffer pH 7.4. The homogenate was centrifuged twice at 2500 g for 10 min. The resulting supernatant was centrifuged at 12,500 g for 20 min. The pellet was washed twice with ice-cold 10 mM Tris-HCl buffer pH 7.4 (1:10 w/v) and centrifuged at 12,500 g for 20 min. The pellet (synaptic membrane, P2) was resuspended in 10 mM Tris-HCl buffer pH 7.4 and freeze-thawed at least three times before been stored at −80°C until used. Protein concentration was determined using the Bradford assay (Bradford, 1976) using bovine serum albumin (BSA) as reference standard.

### [^3^H]Glutamate ([^3^H]Glu) binding

Receptor binding competition assays were performed using rat cortical membranes obtained from Analytical Biological Services, Inc. (Wilmington, DE). The reaction was initiated by adding 100 μg of protein to reaction tubes containing 1 mM of NMDA, KA, and FW for ionotropic glutamate receptors (iGluR) and QA, LCCG-H, DCG-IV, L-AP4 for metabotropic glutamate receptors (mGluR) and 20 nM [^3^H]Glutamate in a final volume of 500 μL of 50 mM Tris HCl/100 mM KCl buffer, pH 7.4. Non-specific binding was determined with the presence of 1 mM non-radioactive glutamate. Total binding was determined in the presence of various concentrations of *C. aurantium* extracts for the receptor competition studies. All samples were incubated on ice for 40 min. The assay was stopped by centrifugation at 6700 g for 30 min at 4°C. The supernatant was removed and pellets were washed twice with 1 mL of ice cold buffer. The pellet was resuspended in 500 μL of buffer. Radioactivity of the samples was quantified in a Beckman LS 6000 counter with 5 ml of EcoLume scintillation fluid. Results are shown as percentage of total binding (AVG ± SEM) (Del Valle-Mojica et al., 2011).

### *C. aurantium* dose response curve

Randomly selected untreated zebrafish were placed in a small chamber (2 cm × 2 cm × 1 cm, length × width × depth) containing 15 mL of a solution of the corresponding *C. aurantium* extract concentration prepared in aquarium water for 1 h. After this, animals were transferred to a clear tank (7.5 cm × 4.5 cm × 6.0 cm, length × width × depth) with a final volume of 100 mL of PTZ (3 mg/mL) prepared in aquarium water by diluting from a 4 M stock solution prepared in deionized water. The time elapsed between exposure to PTZ and loss of coordination and swimming axis after wild jump is considered the seizure latency. Changes in seizure latency caused by the *C. aurantium* extract were measured to determine the effectiveness of the extract. Experiments were recorded using an Olympus FE340 camera.

### *In vivo* extract-receptor interaction experiments

Randomly selected untreated zebrafish were placed for 1 h in a small chamber (2 cm × 2 cm × 1 cm, length × width × depth) containing 15 mL of a solution of the corresponding to the desired concentration of the antagonists: PHCC (mGluR I), EGLU (mGluR II) or CPPG (mGluR III), NBQX (AMPAR), D-AP5 (NMDAR), UBP 301(KAR), and gabazine (GABA_A_R) prepared in aquarium water. Stock solutions of PHCC, EGLU or CPPG, NBQX, D-AP5, and gabazine where prepared in deionized water, UBP 301 stock was prepared in DMSO. For compounds prepared in DMSO, we performed vehicle controls with the correspondent DMSO present as in the antagonist solution. After this, animals were transferred to another small chamber containing 15 mL of a solution of the *C. aurantium* extract prepared in aquarium water for 1 h and then moved to a clear tank (7.5 cm × 4.5 cm × 6.0 cm, length × width × depth) with a final volume of 100 mL with PTZ (3 mg/mL) to induce seizures. Control animals for the antagonists and vehicle controls were transferred from the solution containing the antagonist or vehicle directly into the PTZ. Changes in seizure latency between “Antagonist” and “Antagonist + CA” treated animals were measured to determine possible interactions between the extract and these receptors. Experiments were recorded using an Olympus FE340 camera.

### Statistical analysis

Data was normalized by the control mean and expressed as mean values ± standard error of the mean (SEM) of at least three independent experiments. Difference between the experimental groups was tested for significance using One-Way analysis of variance (ANOVA) followed by the Tukey multiple comparisons test, with *p* < 0.05 calculated using the GraphPad Prism software.

## Results

### Chemical analysis of the *C. aurantium* extract

*C. aurantium* extract was tested for limonene, various flavonoids, and synephrine and related amines. We selected these compounds based on literature describing the characterization of various parts of the plant, characterization of extracts from different extraction methods and the methods and standards available from ChromaDex, Inc. (Castillo et al., 1992; Fugh-Berman and Myers, 2004; Karimi et al., 2012). Table 1 shows the chemical analysis revealed the extract contained 0.107 mg/mL of hesperidin, 0.0115 mg/mL of neohesperidin, and 0.00125 mg/mL neohesperidin dihydrochalcone, trace amounts of nobiletin, rutin, naringin, and individual citrus bioflavonoids although total bioflavonoid concentration was determined at 0.0235 mg/mL. Limonene, naringenin, and hesperetin were not detected in the extract.

**Table 1:** **Chemical characterization of the *C. aurantium* extract**.

**Compound**	**Reporting limit (mg/ml)**	**Quantity detected (mg/ml)**
Limonene	0.019	ND
TOTAL citrus bioflavonoids	Not specified	0.0235
Rutin	0.0012	BRL
Naringin	0.00097	BRL
Naringenin	0.00036	ND
Hesperidin	Not specified	0.107
Neohesperidin	0.0019	0.0115
Neohesperidin dihydrochalcone	0.00043	0.00125
Hesperetin	0.00036	ND
Nobiletin	0.0049	BRL
Synephrine and related amines		
Octopamine HCL	0.000222	BRL
Synephrine	0.00598	BRL
Tyramine HCL	0.00626	ND
Hordenine	0.00297	BRL
Phenylethylamine HCL	0.000316	BRL
Tryptamine	0.000343	BRL

*C. aurantium aqueous leaf extract was analyzed using HPLC and GC for limonene, rutin, naringin, naringenin, hesperidin, neohesperidin, neohesperidin dihydrochalcone, hesperetin, nobiletin, octopamin, synephrine, tyramine, hordenine, phenylethylamine, and tryptamine. Only hesperidin 0.107 mg/mL, neohesperidin 0.0115 mg/mL, and neohesperidin dihydrochalcone 0.00125 mg/mL were able to be quantified*.

*ND, Not detected above reporting limit (RL); BRL, Below reporting limit (compound detected below RL)*.

### *C. aurantium* extracts increases seizure latency in zebrafish

**Figure 1:**
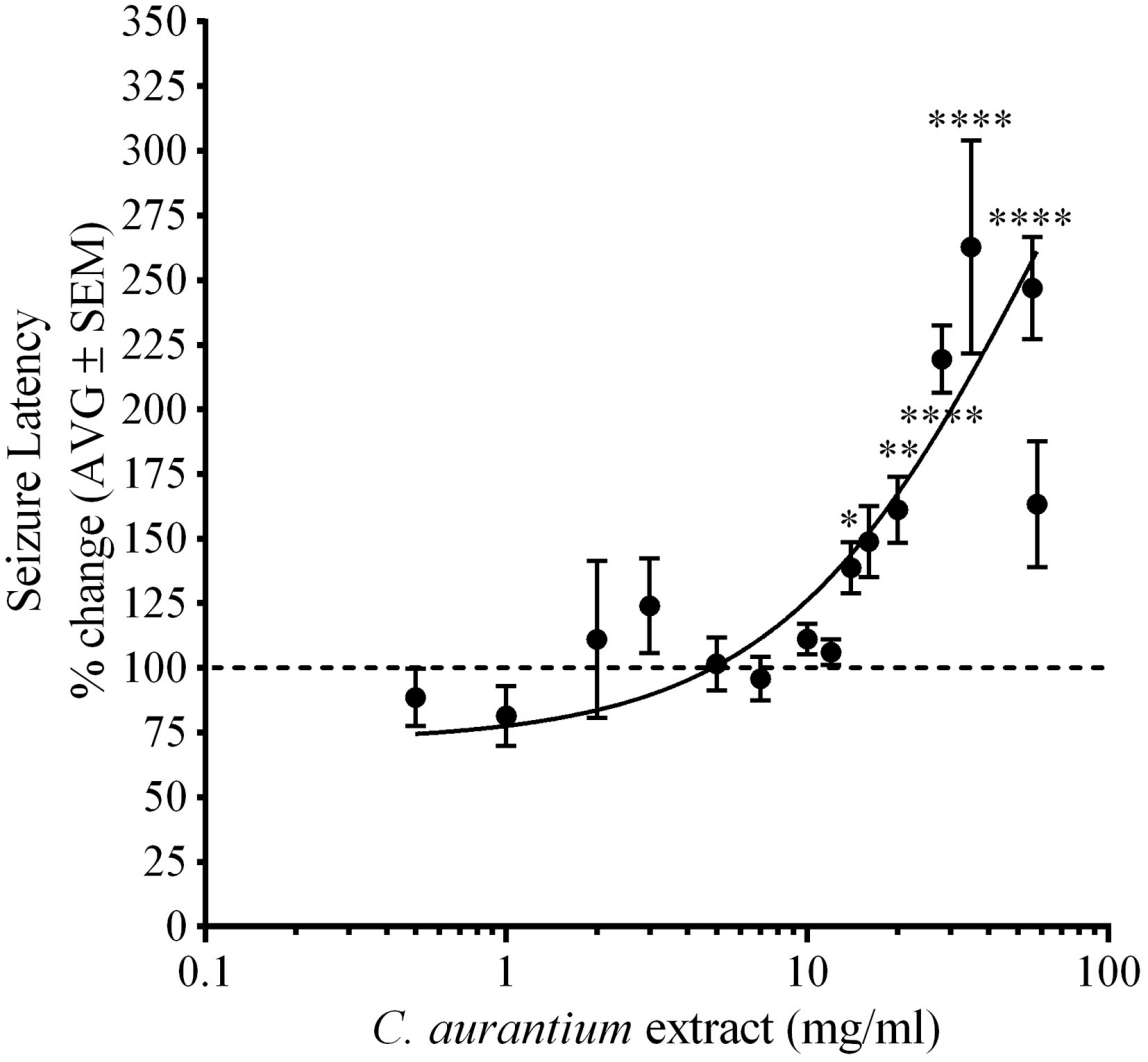
**Dose dependent effect of *C. aurantium* extract on seizure latency to PTZ in adult zebrafish**. Animals were allowed absorption on various concentrations of *C. aurantium* extracts before exposure to PTZ 3 mg/ml. *C. aurantium* extracts at 14, 20, 28, 35, and 56 mg/ml, significantly increased seizure latency compared to naïve animals. The dotted line represents seizure latency of naïve animals. Results are shown as average ± SEM of at least three experiments, *n* > 9. ^*^ vs. naïve *P* < 0.05; ^**^P < 0.01; ^****^P < 0.0001.

Animals were challenged with PTZ following exposure to *C. aurantium* to measure changes on seizure latency. Figure 1 shows the effect of *C. aurantium* extracts in seizure latency was dose dependent. *C. aurantium* extracts at high concentration significantly increased seizure latency to PTZ. *C. aurantium* 56 mg/mL increased seizure latency to 246.9% ± 19.7, *C. aurantium* 35 mg/mL to 262.8% ± 41, *C. aurantium* 28 mg/mL to 219.4% ± 13, *C. aurantium* 20 mg/mL to 161.2% ± 12, and *C. aurantium* 14 mg/mL to 138% ± 9.9 respective to untreated animals. At lower concentrations, 0.5 and 1 mg/mL, *C. aurantium* extracts showed a tendency to reduce seizure latency to PTZ respective to untreated animals. *C. aurantium* 0.5 mg/mL reduced seizure latency to 88.58% ± 11 and *C. aurantium* 1 mg/mL to 81.4% ± 11.6 respective to untreated animals. Concentrations above 35 mg/mL caused a loss of swimming axis during absorption that extended for several minutes after transfer to PTZ. We observed frequent occurrences of toxicity resulting in death during the absorption period at concentrations above 35 mg/mL. For this reason, the number of animals for the concentrations of 35 mg/mL and higher was kept at 5–8 animals to avoid exposing the animals to toxic and lethal doses. We selected *C. aurantium* 28 mg/mL for the selectivity assays because we observed the highest reproducible effect without constant signs of toxicity. The increase in seizure latency to 219.4% ± 19.7, was equivalent to a change in seizure latency from an average of 72.71% ± 3 for naïve animals to an average of 168% ± 17.5 for animals treated with *C. aurantium* 28 mg/mL.

### GABA_A_ receptors are not responsible for the effect of *C. aurantium* extracts

**Figure 2:**
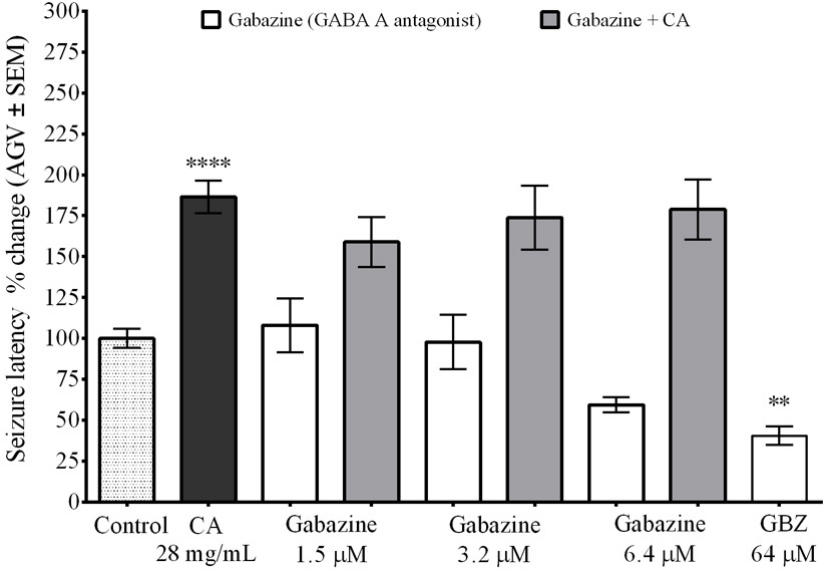
**Interaction between *C. aurantium* extract and GABA_A_ receptors**. Fish were allowed 1 h absorption in various concentrations of selective GABA_A_ receptor antagonist gabazine, followed by 1 h absorption on *C. aurantium* extract at 28 mg/mL prior exposure to PTZ 3 mg/mL. Gabazine alone had no significant effect when compared to the control animals. Neither when administered before *C. aurantium* 28 mg/mL extract. Results are shown as average ± SEM of at least three experiments, *n* > 12. ^**^ vs. Naive *P* < 0.01; ^****^P < 0.0001.

We used the GABA_A_ receptor antagonist gabazine (GBZ) to determine if the change in seizure latency caused by the *C. aurantium* extract was caused by an interaction with GABA_A_ receptors (Figure 2). We used three different concentrations of gabazine, 1.5, 3.2, and 6.4 μM. Seizure latency with respect to untreated animas after exposure to gabazine 1.5 μM was 108% ± 16, for 3.2 μM was 97.7% ± 17, and for 6.4 μM was 59.4% ± 5. An additional concentration of 64 μM was tested as positive control for the activity of gabazine. This dose significantly reduced seizure latency to 40% ± 6 respective to untreated animals. Gabazine administration before exposure to the *C. aurantium* 28 mg/mL extract, reduced seizure latency to 159 ± 15% (GBZ 1.5 μM), 173 ± 20% (GBZ 3.2 μM), and 178.9% ± 18 (GBZ 6.4 μM) respective to untreated animals. These changes represented a reduction of 16% (GBZ 1.5 μM), 7% (GBZ 3.2 μM), and 4% (GBZ 6.4 μM) on the effect on seizure latency caused by the *C. aurantium* 28 mg/mL extract but this changes were not statistically significant, eliminating GABA_A_ receptors as mediators of the effect of *C. aurantium* extracts.

### *C. aurantium* extract caused a dose dependent change on [^3^H]Glutamate binding to glutamate receptor

**Figure 3:**
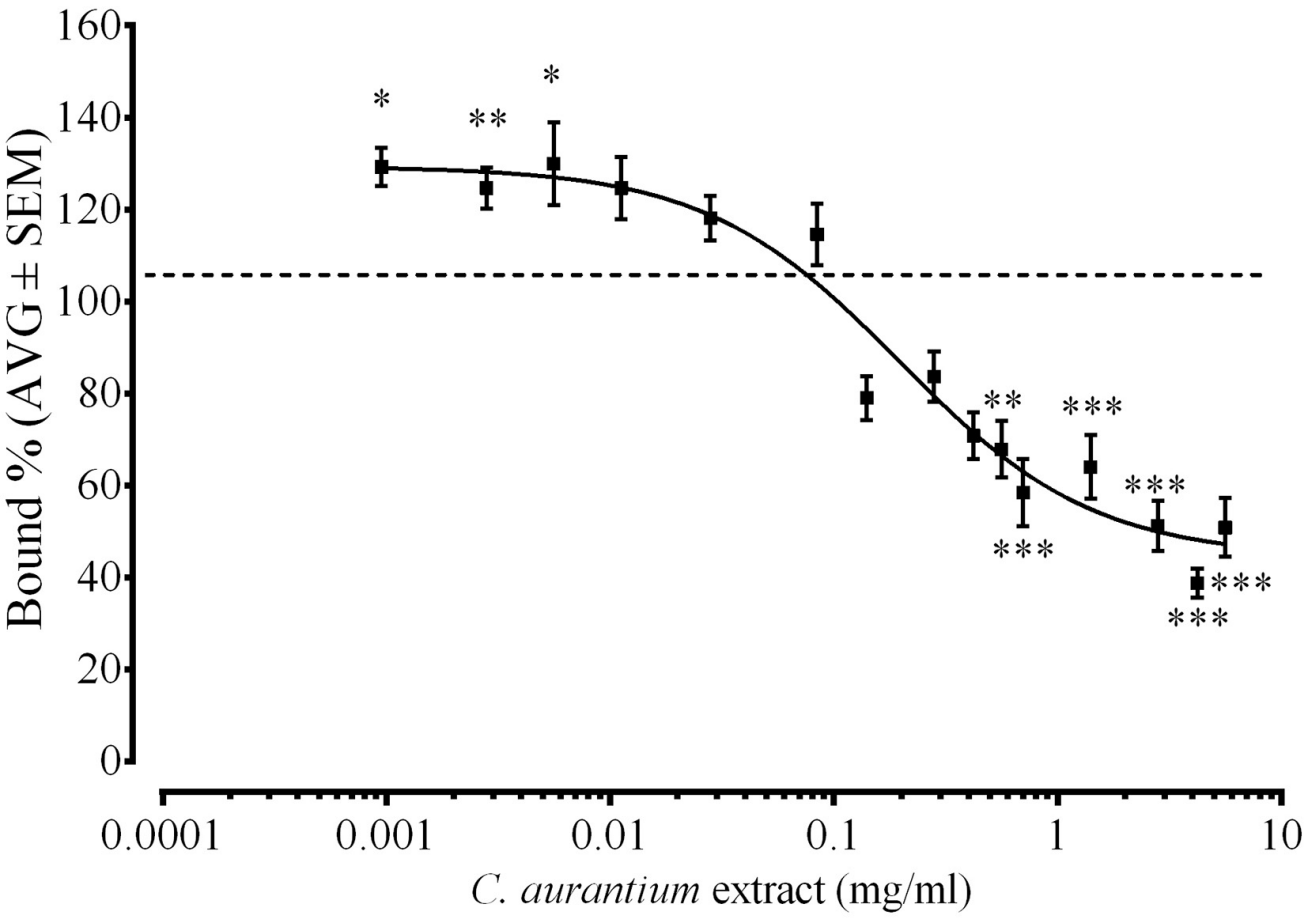
**Effect of *C. aurantium* extract on [^3^H]Glu binding**. Synaptic membranes were incubated with different concentrations of *C. aurantium* in the presence of 20 nM [^3^H]Glu. *C. aurantium* extract reduced [^3^H]Glu binding at concentrations ranging from 0.42 to 5.60 mg/mL and increased [^3^H]Glu binding at concentrations ranging from 0.000952 to 0.0056 mg/mL. Results are shown as percentage of total binding ± SEM of three experiments. ^*^ vs. Total *P* < 0.05; ^**^P < 0.01; ^***^P < 0.001.

We used a radioligand binding assay to determine if the *C. aurantium* extract binds to glutamate receptors. Figure 3 shows the dose dependent interaction between *C. aurantium* extracts and glutamate receptors. At low concentrations (0.001, 0.003, and 0.0056 mg/mL), *C. aurantium* extracts significantly increased [^3^H]Glu binding to glutamate receptors to 129.3% ± 4 bound, 125% ± 4.5 bound, and 130% ± 9 bound, respectively. This represents a 26–36% increase in [^3^H]Glu binding to glutamate receptors. This effect is associated to the presence of allosteric modulators that can alter the binding affinity of the radioactive ligand increasing on and off target binding (Leysen et al., 2014). On the other hand, at high concentrations (0.56–5.6 mg/mL), *C. aurantium* extracts significantly reduced [^3^H]Glu binding from 67.8% ± 6 to 38.8% ± 3 bound. This represents a 30–60% reduction in [^3^H]Glu binding to glutamate receptors. We selected the concentration of *C. aurantium* 1.4 mg/mL for the *In vitro* interaction assays because this concentration caused approximately 60% displacement on [^3^H]Glu binding.

### *C. aurantium* reduced [^3^H]Glu binding to NMDA and mGLuR II

**Figure 4:**
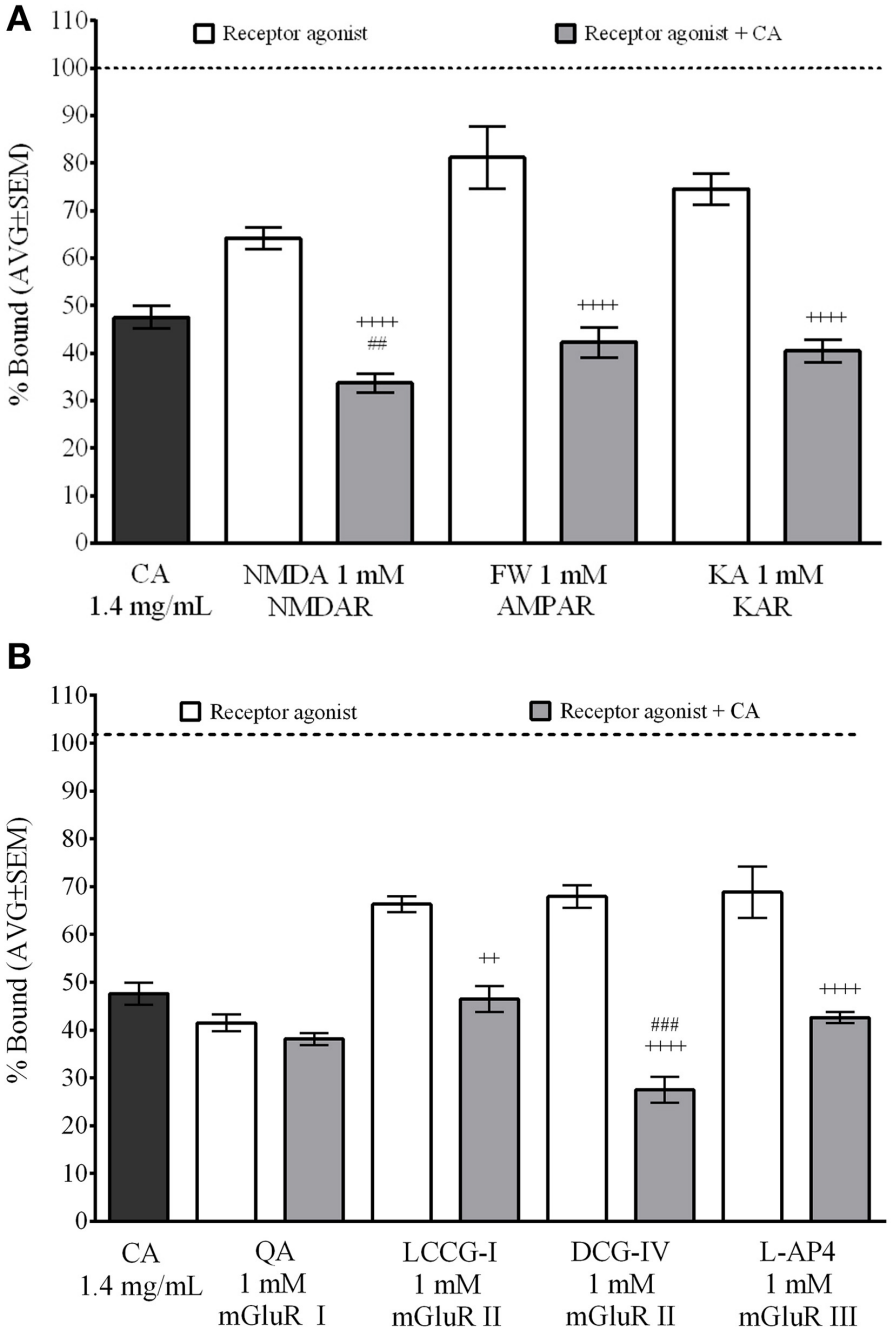
**Selective interaction of *C. aurantium* extract with glutamate receptors *In vitro***. Synaptic membranes were incubated with *C. aurantium* extract at 1.4 mg/mL in the presence of 1 mM of agonists for iGluR's o mGluR's and 20 nM [^3^H]Glutamate. For these experiments, nonspecific binding was 22% of total binding (represented with a dotted line). All treatments significantly reduced [^3^H]Glu binding, when compared with Total binding. **(A)** *C. aurantium* 1.4 mg/mL reduced ligand binding in the presence of ionotropic glutamate receptor agonist for NMDA, AMPA, and KA receptors compared to the agonist alone. **(B)** C. aurantium 1.4 mg/mL reduced ligand binding in the presence of metabotropic glutamate receptor agonist for mGluR II (LCCG-I, DCG-IV) and mGluR III (L-AP4) when compared to the agonist alone. Results are shown as percentage of total binding ± SEM of three experiments. ^++^ vs. Agonist *P* < 0.01; ^++++^*P* < 0.0001. ^##^ vs. CA1.4 mg/ml *P* < 0.01; ^###^*P* < 0.001.

We used the same radioligand binding assays in the presence of selective glutamate receptor agonist to identify the specific receptors *C. aurantium* extract binds to (Figure 4). Assay set up included non-specific binding control (off target [^3^H]Glu binding, approximately 10–20% of total binding) and total binding control to validate the selectivity of the radio-ligand (not shown). Also, we performed controls for water, extract alone and agonist alone to establish the baseline displacement used to compare the effect of the mixture of extract and agonist. Receptor agonist were used at 1 mM, a concentration that allows us to determine a ligand selective effect on [^3^H]Glu binding. *C. aurantium* 1.4 mg/ml reduced [^3^H]Glu binding to 47.6% ± 2, this represent a 52% reduction in [^3^H]Glu binding to glutamate receptors. Figure 4A shows ionotropic glutamate receptor (iGluR) agonist for NMDA, AMPA (FW), and KA receptors, significantly reduced total [^3^H]Glu binding to 64.2% ± 2, 81.2% ± 7, and 74.6% ± 2, respectively. These represents a 36%, 19%, and 25% reduction in [^3^H]Glu binding, respectively. In the presence of both, the receptor agonist and *C. aurantium* extract at 1.4 mg/mL, total [^3^H]Glu binding was significantly reduced for NMDA receptors to 33.7% ± 2, for AMPA receptors to 42.3% ± 3 and for Kainate receptors to 40.4% ± 2. This represented a significant 47% (NMDA), 48% (AMPA), and 46% (KA) reduction in [^3^H]Glu binding when compared to the agonist alone. When compared to the extract alone, it represented a significant reduction for NMDA receptors of 29%, but not for AMPA (11% reduction) and KA receptors, (15% reduction).

For the metabotropic glutamate receptors (mGluR) (Figure 4B), QA (mGluR I), LCCG-I and DCG-IV (mGluR II) and L-AP4 (mGluR III) significantly reduced total [^3^H]Glu binding to 41.5% ± 2, 66.3% ± 2, 67.9% ± 2, and 68.9% ± 5. These represented a respective 58%, 34%, 32%, and 31% reduction in total [^3^H]Glu binding. When both, agonist and *C. aurantium* 1.4 mg/mL extracts were present, [^3^H]Glu binding was significantly reduced for LCCG-I and DCG-IV and L-AP4 to 46.5% ± 2, 27.5% ± 3, and 42.6% ± 1, respectively. These represented a significant reduction in [^3^H]Glu binding of 30%, 60%, and 38% when compared to the agonist alone. When compared to the extract alone, it represented a significant reduction in [^3^H]Glu binding of 42%, but not for LCCCG-I (2% reduction) or L-AP4 (10.6% reduction). For QA, [^3^H]Glu binding was reduced to 38.1 ± 1. This represented an 8% reduction when compared to the agonist and 19% reduction when compared to the extract but these changes were not statistically significant.

### NMDA receptor antagonist D-AP5 inhibits the effect of *C. aurantium* on PTZ induced seizures in zebrafish

**Figure 5:**
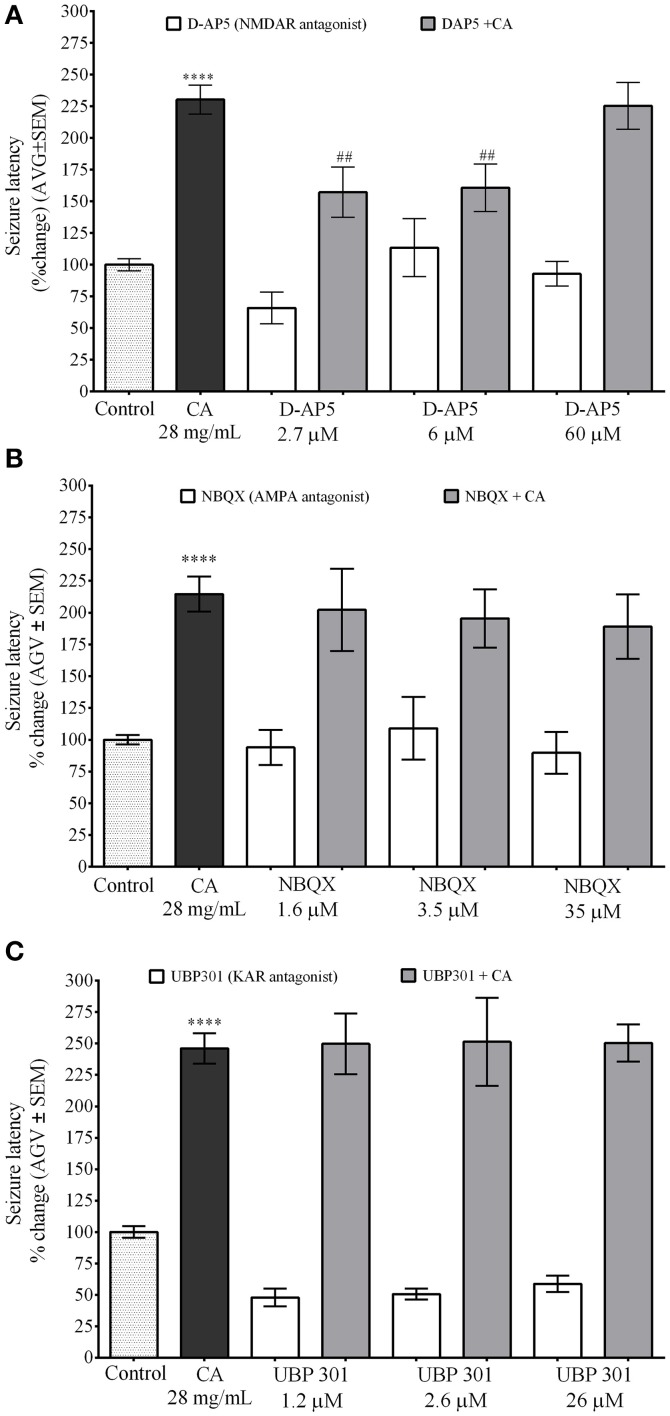
**Selective *In vivo* interaction of *C. aurantium* extract with iGluR**. Fish were allowed 1 h absorption of various concentrations of selective iGluR antagonist D-AP5 (NMDAR), NBQX (AMPA), UBP 301 (KAR) followed by 1 h absorption on *C. aurantium* extract at 28 mg/mL prior exposure to PTZ 3 mg/mL. **(A)** D-AP5 2.7 and 6 μM prior exposures to *C. aurantium* extract significantly reduced seizure latency when compared to *C. aurantium* alone. **(B)** Exposure to NBQX prior exposures to *C. aurantium* extract showed a tendency to reduce seizure latency when compared to *C. aurantium* alone. **(C)** Exposure to UBP 301 prior exposures to *C. aurantium* extract had no significant effect on seizure latency when compared to *C. aurantium* alone. Results are shown as average ± SEM of at least three experiments, *n* > 12. ^****^ vs. Naive *P* < 0.0001, ^##^ vs. *C. aurantium* 28 mg/mL *P* < 0.01.

These experiments where performed using a concentration of iGluR antagonist with no significant effect on seizure latency. NMDA receptor antagonist D-AP5 was used at concentrations of 2.7 μM (seizure latency 66% ± 12), 6 μM (seizure latency 113.4% ± 23), and 60 μM (seizure latency 92.9% ± 10). Seizure latency of the *C. aurantium* extract was 230.3% ± 12. Exposure to D-AP5 2.7 μM and 6 μM before exposure to the *C. aurantium* 28 mg/mL extract significantly reduced seizure latency to 157.2% ± 20 and 160.7% ± 19, respectively. This represented a significant 32% and 30% reduction on seizure latency compared to *C. aurantium* 28 mg/mL alone (Figure 5A). Exposure to D-AP5 60 μM before exposure to the extract reduced seizure latency to 92.8% ± 9.6 but this effect was not statistically significant when compared to the effect of the extract alone (Figure 5A).

AMPA receptor antagonist NBQX was used at concentrations of 1.6 μM (seizure latency 94% ± 13.77), 3.5 μM (seizure latency 109% ± 25), and 35 μM (seizure latency 89.7% ± 16.5). Seizure latency of the *C. aurantium* extract was 214.6% ± 13.7. Exposure to NBQX before *C. aurantium* had no significant effect in seizure latency when compared to *C. aurantium* 28 mg/mL alone (Figure 5B) (seizure latencies 202.2% ± 32.4, 195.5% ± 23, 189.1% ± 25.3, respectively).

Lastly, the concentrations selected for the kainate receptor antagonist UBP 301 were 1.2 μM (seizure latency 47.8% ± 7), 2.5 μM (seizure latency 50.5% ± 4), and 25 μM (seizure latency 58.7% ± 6). Seizure latency of the *C. aurantium* extract was 246.1% ± 12. Pre exposure to UBP 301 prior exposure to *C. aurantium* extracts had no effect on seizure latency when compared to *C. aurantium* 28 mg/mL alone (Figure 5C) (seizure latencies: 249.7% ± 24, 251.4% ± 35, and 250.4% ± 14.9, respectively). Since UBP 301 stock solution was prepared in DMSO following solubility indications, DMSO controls were performed in parallel (see Supplementary Figure 1). The corresponding concentrations of DMSO in the antagonist solution were 0.023% DMSO on the UBP 301 1.2 μM solution (seizure latency 95.6% ± 17), 0.051% DMSO on the UBP 301 2.6 μM (seizure latency 59.5% ± 7) solution and 0.51% DMSO on the UBP 301 26 μM solution (seizure latency 78.82% ± 22). DMSO alone had no significant effect on seizure latency to PTZ, nor did it alter the effect of C*. aurantium* on seizure latency (221.9% ± 0.5, 237% ± 30.2, 189.7% ± 31.7).

### mGLuR I antagonist PHCCC and mGuR II antagonist EGLU inhibit the effect of *C. aurantium* on PTZ induced seizures in zebrafish

**Figure 6:**
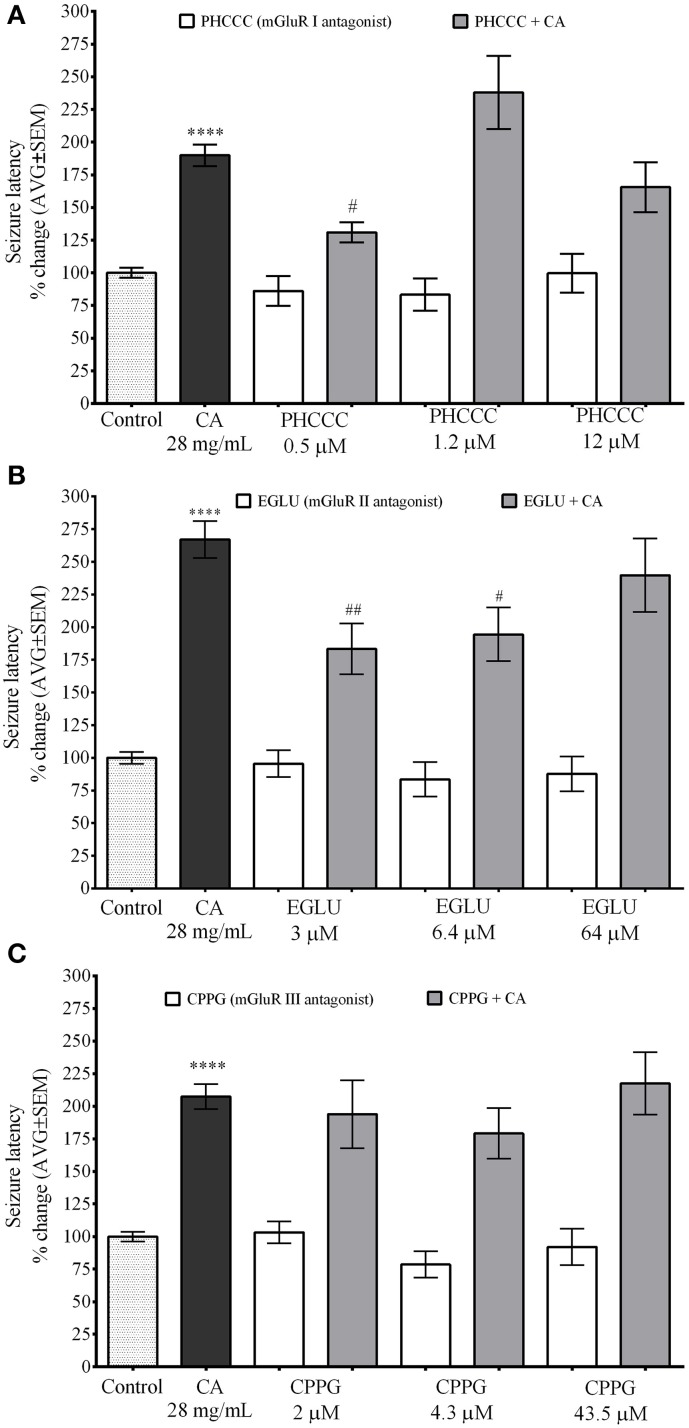
**Selective *In vivo* interaction of *C. aurantium* extract with mGluR**. Fish were allowed 1 h absorption of various concentrations of selective mGluR antagonist PHCC(mGluRI), EGLU(mGluRII), CPPG(mGLURII) followed by 1 h absorption on *C. aurantium* extract 28 mg/mL prior exposure to PTZ 3 mg/mL. **(A)** PHCC at 0.5 μM significantly reduced seizure latency by 32% and at 1.2 μM significantly increased seizure latency by 38% when compared to *C. aurantium* alone. **(B)** EGLU 3 and 6.4 μM, significantly reduced the increase in seizure latency caused by treatment with *C. aurantium* extract by 33 and 29%, respectively. **(C)** mGluR III antagonist CPPG had no effect on the changes in seizure latency caused by exposure to *C. aurantium* extract. Results are shown as average ± SEM of at least three experiments, *n* > 12. ^****^ vs. Naive *P* < 0.0001, ^#^ vs. *C. aurantium* 28 mg/ml *P* < 0.05; ^##^*P* < 0.01.

Figure 6 demonstrate the selectivity of the effects of *C. aurantium* extracts on seizure latency in zebrafish. These experiments were also performed using a concentration of mGluR antagonist with no significant effect on seizure latency. Seizure latency of the *C. aurantium* extract was 189.9% ± 13.7. We used PHCCC at 0.5 μM (seizure latency 86.1% ± 11), 1.2 μM (seizure latency 83.2% ±12.3), and 12 μM (seizure latency 99.6% ± 15). Pretreatment with PHCCC (mGluR I antagonist) 0.5 μM prior exposure to *C. aurantium* extracts significantly reduced seizure latency to 130.9% ± 7.8 compared to the extract alone. This represented a 31% reduction in the effect of the *C. aurantium* 28 mg/mL extract. Pretreatment with PHCCC 1.2 μM prior exposure to *C. aurantium* extracts increased seizure latency to 238% ± 28 but this change was not statistically significant when compared to the effect of the extract alone. Pretreatment with PHCCC 12 μM had no significant effect on seizure latency when compared to either the antagonist or the extract alone (Figure 6A).

mGluR II antagonist EGLU at 3 μM had a seizure latency of 95.48% ± 10.2, at 6.4 μM of 83.5% ± 13.1, and at 64 μM of 87.8% ± 13.3. Seizure latency of the *C. aurantium* extract was 267.1% ± 14. Pretreatment with EGLU 3 μM and 6.4 μM prior exposure to *C. aurantium* extract significantly reduced seizure latency to 183.3% ± 19.4 and 194.4% ± 20.5, respectively. These represented a 31% (EGLU 3 μM) and 27% (EGLU 6 μM) reduction in the effect of the *C. aurantium* 28 mg/mL extract. Pretreatment with EGLU 64 μM before the extract had no significant effect on seizure latency when compared to the extract alone (seizure latency 239.8% ± 28.3).

We used CPPG (Figure 6C), a selective mGluR III receptor antagonist at concentrations of 2 μM (seizure latency 103.2% ± 8.5), 4.3 μM (seizure latency 78.6% ± 10), and 43.5 μM (seizure latency 92.1% ± 14). Pre-exposure to CPPG prior the *C. aurantium* extract had no significant effect on seizure latency when compared to the extract alone (seizure latencies 193.9% ± 26.6, 179.1% ± 19.5, and 217.5% ± 24).

## Discussion

*C. aurantium* is extensively used as a dietary supplement for weight loss—although efficacy has not been established. Research on anxiolytic and anticonvulsant properties has focused on the properties of the essential oils of the peel or the product of organic extractions made from dried leaves (Carvalho-Freitas and Costa, 2002; Pultrini et al., 2006; Alvarado-Guzmán et al., 2009).

To our knowledge, this is the first time that an aqueous extract made from the leaves of the *C. aurantium* tree has been studied, giving insight to the pharmacological properties of a preparation commonly used by patients, particularly in Puerto Rico. The use of adult zebrafish to screen for activity in natural products is also a relatively novel approach since the use of the larvae is more popular for this kind of studies and the adult is only starting to gain popularity for this applications.

We were able to establish this aqueous preparation can increase seizure latency to PTZ in adult zebrafish. This is consistent with findings on mice obtained using the essential oils from the peel of the fruit (Carvalho-Freitas and Costa, 2002). This effect was dose dependent, and reached maximum effectiveness around 28 mg/mL with higher concentrations increasing the frequency of toxicity in the form of ataxia or loss of motor coordination caused by neurotoxicity (Kalueff et al., 2013). We tested other members of the citrus genus like *C. maxima* (grapefruits) and from a hybrid of *C. aurantium* and *C. sinensis* (see Supplementary Figure 2) for anticonvulsant properties but it appears this is a unique property of the *C. aurantium* tree.

We established our extract contains detectable amounts of the flavonoids hesperidin, neohesperidin, and neohesperidin dihydrochalcone and trace amounts of other flavonoids like naringin, rutin and nobiletin, and synephrine alkaloids like octopamine, synephrine, hordenine, phenylethylamine, and tryptamine. This is consistent with literature showing extracts from the peel of the fruit, and the flower bloom contain similar profile of compounds although we only found trace amounts of synephrine alkaloids and these are highly abundant in this other parts of the plant (Fugh-Berman and Myers, 2004; Liu et al., 2008). These compounds have been attributed with a range of properties. For example, naringin is shown to cross the blood brain barrier (Ameer et al., 1996; Zbarsky et al., 2005) and to have antioxidant, anti-inflammatory, antihypercholesterolemic, antihypertensive, neuroprotective (Golechha et al., 2011; Chanet et al., 2012; Karimi et al., 2012), and anticonvulsant properties—it increases seizure latency to kainic acid administration (Golechha et al., 2011). Naringenin and hesperetin activate the peroxisome proliferator-activated receptor (PPAR) and up-regulating adiponectin expression in adipocytes giving them antiatherogenic properties (Liu et al., 2008). Hesperidin, neohesperidin, and neohesperidin dihydrochalcone, the most abundant species detected, are attributed antioxidant (Kumar et al., 2013; Hu et al., 2014) anti-inflammatory (Hamdan et al., 2014; Ho and Kuo, 2014), vasopresive and antiplatelet properties (Majumdar and Srirangam, 2009) and may be responsible for the anticonvulsant properties of the *C. aurantium* extract. A broader analysis could have revealed additional compounds present in the extract giving a more comprehensive characterization of the extract.

It is important to understand the possible molecular mechanisms behind the changes in seizure latency caused by this citrus extract. GABA and glutamate are the mayor neurotransmitters in the brain and are implicated in the pathophysiology of epilepsy. We used GABA_A_ receptor antagonist gabazine to rule out an interaction with the GABA binding site and focused on glutamate receptors. The excitatory neurotransmitter glutamate has been implicated in early changes that lead to the initiation of hyperactivity, but also to the amplification and spread of the excitatory hyperactivity (Wong et al., 1999; Chapman, 2000; Moldrich et al., 2003; Ure et al., 2006) acting thru two main families of receptors, the ionotropic (iGluR) and metabotropic glutamate receptors (mGluR)(Ozawa et al., 1998). The iGluR's are a family of ligand gated ion channels comprised by NMDA, AMPA, and Kainate receptors that modulate neuronal excitability altering ion conductance. The mGluR's are a family of G-protein coupled receptors comprised by subtypes 1 thru 8 that modulate cellular excitability by activation of second messenger systems and intracellular signaling cascades. Both type of receptors have been implicated in the etiology of different types of seizures (Bradford, 1995; Moldrich et al., 2003; Ure et al., 2006).

We used an *In vitro* ligand binding assay based on binding of [^3^H]Glu to glutamate receptors in a preparation of rat cortical membranes to demonstrate a dose dependent interaction between *C. aurantium* extracts and glutamate receptors (Figure 2). High concentrations of the extract significantly reduced [^3^H]Glu binding suggesting the extract and the radio-ligand bind and compete for the same binding site. This findings are consistent with that observed *In vivo* where at high concentrations increased seizure latency. Low extract concentrations significantly increased [^3^H]Glu binding. In ligand binding assays, allosteric modulators can alter the binding affinity increasing the binding of the radioactive ligand to on and off target binding sites (Leysen et al., 2014). This suggests at this concentrations the effect of a compound behaving as an allosteric modulator could be prevailing and this could correlate with a slight tendency of lower extract concentrations to reduce seizure latency. Both observations must be studies in further detail to better understand the effect of the *C. aurantium* extract and its interaction with glutamate receptors.

Furthermore, we used selective receptor agonist at concentrations that allowed us evaluate a “ligand-selective” effect on [^3^H]Glu binding to determine the selectivity of this interaction. We identified interactions between the extract and the iGluR's NMDA, AMPA, and kainate receptors, and the mGluR's groups II and III, indicated by a significant reduction in [^3^H]Glu binding when *C. aurantium* was combined with the receptor agonists greater than the one caused by the agonist alone. This reduction was significantly lower when compared with *C. aurantium* for NMDA and DCG-IV, pointing to a stronger interactions with these two receptors. For the mGluR II, we used both DCG-IV and LCCG-I because DCG-IV has been shown to interact with NMDA receptors and LCCG-I is considered to be more selective for mGluR II although it can bind to other mGluR's at micromolar concentrations (Schoepp et al., 1999; Nicoletti et al., 2011). We saw a greater displacement of [^3^H]Glu binding with both DCG-IV and LCCG-I. Interaction with multiple receptors suggest the extract could be interacting with a common binding site in these receptors—the glutamate binding site. Additional experiments using radiolabeled selective agonists for each receptor could help refine our findings.

We used our zebrafish model of PTZ induced seizures and the *C. aurantium* extract at 28 mg/mL to validate these interactions. We selected this concentration because it gave us the maximal protective effect with very limited toxicity events contrary to the higher concentrations that showed frequent toxicity. We used selective receptor antagonists at concentrations that did not caused a significant change in seizure latency to PTZ in the zebrafish. NMDA receptor antagonist D-AP5 significantly reduce the effect of *C. aurantium* extract on seizure latency. These suggest NMDA receptors may be involved in the mechanism of action of *C. aurantium. In vitro* results suggest our extract might be an antagonist at the glutamate binding site. *In vivo* results suggest our extract could be acting as a receptor antagonist or as a partial agonist resulting in reduced excitability because D-AP5 2.7 and 6 μM suppressed the effect of the extract and with 2 receptor antagonists at NMDA receptors you would expect to see an increase of the anticonvulsant effect. According to the literature, D-AP5 does not cross the blood brain barrier (BBB) in rodents (Kapur and Lothman, 1990). The blood brain barrier of larvae zebrafish is described to possess permeability properties to mammals (Jeong et al., 2008) but it is not described in adult zebrafish. Although we observed a reduction on seizure latency after exposure to D-AP5, this could be due to changes in BBB permeability caused by NMDA receptor antagonist (Neuhaus et al., 2011). To address this, we used the noncompetitive NMDA receptor antagonist (MK801) that binds to the ion channel of the receptor (Supplementary Figure 4). MK801 3.5 and 35 μM significantly increased the effect of the extract. Another mechanism that was not evaluated could involve the glycine binding site on NMDA receptors. AMPA receptor antagonist had no significant effect on the seizure latency of the *C. aurantium* extract. KA receptor antagonist UBP 301 alone slightly reduced seizure latency. This reduction was not significantly different than that caused by its DMSO vehicle suggesting this slight reduction could result from the DMSO and not the UBP301. Despite this, the efficacy of the extract was maintained. This should be investigated further in order to accurately determine if KA receptors have a role in the mechanism of *C. aurantium*.

For the metabotropic glutamate receptors, mGluR I antagonist PHCC reduced the effect of *C. aurantium* extracts in seizure latency to PTZ. This receptors are associated with increased neuronal excitability (reviewed by Ure et al., 2006). This could mean, the *C. aurantium* extract could be acting as a receptor antagonist. This interaction must be evaluated in further detail since PHCC 0.5 μM significantly reduced the effect of the *C. aurantium* extract but at 1.2 μM we saw a slightly increased seizure latency over that of *C. aurantium* alone and 12 μM slightly reduced seizure latency. This could reflect a non-monotonic dose response relationship for PHCC or changes in the affinity or selectivity at the higher concentrations. Additional experiments using more selective receptor antagonists including selective antagonists for receptors subtypes could help clarify this findings.

mGluR's II and III are associated with a reduction in neuronal excitability (reviewed by Ure et al., 2006). mGluR II antagonist EGLU at 3 and 6.4 μM, significantly reduced the effect of the *C. aurantium* extract suggesting it is acting as an agonist on this receptors. At the higher concentration, we saw the effect of the extract returned to its basal level suggesting at these concentrations EGLU could be having of target effects. Further experiments with subtype specific antagonist could help refine these findings. mGluR III receptor antagonist CPPG had no effect on the increase on seizure latency caused by the *C. aurantium* extract.

Taken together, these results demonstrate that the aqueous extract made from the leaves of the *C. aurantium* tree possess neuroactive properties; specifically, they alter the seizure latency to PTZ. In addition, we demonstrated an interaction with NMDA and mGluR's I and II in zebrafish as predicted by pharmacological assays. Our results suggest that *C. aurantium* extracts may also be relevant to modulate affective components of behavior such as anxiety, where mGluR's have a crucial role in the mechanism of the disease. In fact, preliminary experiment using the open field test adapted for zebrafish showed anxiolytic properties (data not shown). Also, it would be important to evaluate if this aqueous preparation can protect against cognitive impairment, supporting the idea that its use as adjuvant therapy can also contribute to reduce the sequelae caused by seizure and also some AED's like phenobarbital and topiramate (Cramer et al., 2010).

This result also highlight the relevance of aqueous extractions to screen crude extracts made from natural products. We recognize theses preparations pose a challenge when evaluating pharmacokinetic and pharmacodynamic interactions but we believe there is potential in them. Organic solvents are the most common solvents used in the extraction of natural products. Since aqueous and organic solvents produce extracts with different composition profile, screening them in parallel, at least at early stages of research, could increase the success rate of the screening process. Another important step to solidify our work would be to repeat this experiment using the product of an organic extraction to compare the composition and effect of both preparations. Also, our experiments were performed using only one receptor antagonist per receptor types. Using additional selective receptor antagonist and using selective antagonist for mGluR subtypes would be important to refine our findings.

In addition, we plan to explore the anticonvulsant properties of hesperidin, the most abundant compound found in our extract to determine if the anticonvulsant effect of the extract could be attributed to this individual compound or if this property results from the mixture of compounds. This would be of great value since recent experiments show chronic oral administration of hesperidin can reduce the course of kindling with PTZ in mice, reduced the levels of oxidative stress indicators which play an important role in the pathophysiology of epilepsy (Sudha et al., 2001; Chuang, 2010; Waldbaum and Patel, 2010; Kumar et al., 2013).

Our results highlight the importance of naturally occurring compounds in the treatment of epilepsy and other neurological conditions. With our work, we expect to help draw more attention to the potential of natural product as a therapeutic options either instead of or in combination with conventional medicine in a not so distant future considering the use of natural products is growing more popular (Harris et al., 2012).

## Author contributions

Coral Rosa-Falero and José G. Ortiz took part on the conception and design of the experiments, drafting and revising the manuscript for content and final approval of its content. Coral Rosa-Falero took part in also the execution of the experiments and analysis of the data. Stephanie Torres-Rodríguez and Jeffrey Sosa participated in the design, execution and analysis of the *In vitro* experiments. Claudia Jordán, Rígel Licier, Yolimar Santiago, Zuleyma Toledo, Marely Santiago, and Kiara Serrano took part in the execution and analysis of the *In vivo* experiments. All of the authors participated in the revision of the manuscript draft and approval of the content and agree to be accountable for the accuracy and integrity of the work.

### Conflict of interest statement

The authors declare that the research was conducted in the absence of any commercial or financial relationships that could be construed as a potential conflict of interest.

## Abbreviations

CA: Citrus aurantium
[3H]Glu: [3H]Glutamate
AMPA: Alpha-amino-3-hydroxy-5-methylisox-azole-4-propionic acid
BSA: Bovine serum albumin
CPPG: (RS)-α-Cyclopropyl-4-phosphonophenylglycine.
DCG-IV: (2S,2′3R,3′R)-2-(2′,3′-Dicarboxycyclopropyl)glycine
EGLU: (2S)-a-Ethylglutamic acid
FW: Fluorowillardiine
iGluR: Ionotropic glutamte receptor
KA: Kainic acid
L-AP4: L-(+)-2-Amino-4-phosphonobutyric acid
LCCG-I: (2S,1′S,2′S)-2-(Carboxycyclopropyl)glycine
mGluR I: Group I of the metabotropic glutamate receptor (1,5)
mGluR II: Group II of the metabotropic glutamate receptors (2,3)
mGluR III: Group III of the metabotropic glutamate receptors (4,6,7,8)
NMDA: N-methyl-D-aspartic acid
PHCCC: N-Phenyl-7-(hydroxyimino)cyclopropa[b]chromen-1a-carboxamide
PTZ: Pentylenetetrazole
QA: quisqualic acid: (2S)-2-amino-3-(3,5-dioxo-1,2,4-oxadiazolidin-2-yl) propanoic acid)
D-AP5: D-(-)-2-Amino-5-phosphonopentanoic acid
NBQX: 2,3-Dioxo-6-nitro-1,2,3,4-tetrahydrobenzo[f]quinoxaline-7-sulfonamide
UBP 301: (αS)-α-Amino-3-[(4-carboxyphenyl)methyl]-3,4-dihydro-5-iodo-2,4-dioxo-1(2H)-pyrimidinepropanoic acid
ACN: Acetonitrile
MeOH: Methanol
GBZ: gabazine.
