# Supplementary material for: Citrus aurantium increases seizure latency to PTZ induced seizures in zebrafish thru NMDA and mGluR's I and II
Source: Front Pharmacol. 2015 Feb 13;5:284. doi: 10.3389/fphar.2014.00284 (PMC4327740; doi:10.3389/fphar.2014.00284)
Supplement: Supplementary file 1 [file Image1.PDF]

Supplementary Material

Modulation of PTZ induced seizures by *Citrus aurantium* in zebrafish: role of NMDA and metabotropic glutamate receptors.

Coral Rosa-Falero[1*], Stephanie Torres-Rodríguez[1], Rigel Licer[1], Yolimar Santiago[1], Zuleima Toledo[1], Marelys Santiago[1], Kiara Serrano[1], Claudia Jordán[1], Jeffrey. Sosa[2], and Jose G. Ortiz[1]

[1]Neuropharmacology Laboratory, Pharmacology and Toxicology Department, University of Puerto Rico-Medical Sciences Campus, San Juan, Puerto Rico
[2]RISE Program, Universidad del Este, Carolina, Puerto Rico

*** Correspondence:** Coral Rosa-Falero, [1]Neuropharmacology Laboratory, Pharmacology and Toxicology Department, University of Puerto Rico-Medical Sciences Campus, P.O. Box 365067, San Juan, 00936-5067, Puerto Rico.
coral.rosa.falero@gmail.com

1. Supplementary Data

1.1. Supplementary Figure 1

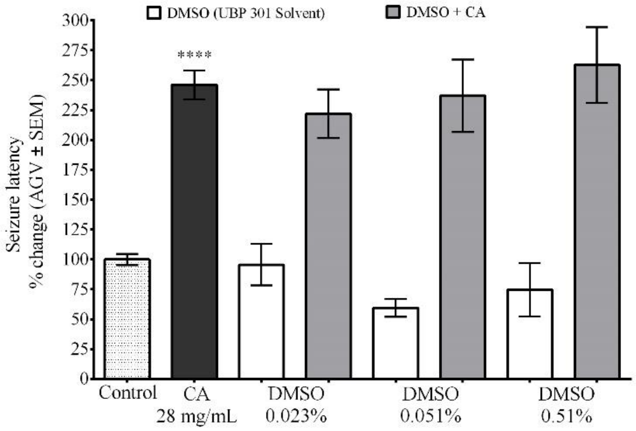

Supplementary Figure 1. DMSO as solvent for UBP 301 and its effects on seizure latency. Fish were allowed 1h absorption in DMSO concentrations equivalent to those in the antagonist solution. This was carried out in parallel with the UBP 301 experiments. DMSO 0.023% corresponds to the DMSO concentration in UBP 301 1.2µM, DMSO 0.051% corresponds to UBP 301 2.6 µM and DMSO 0.51% corresponds to UBP301 26µM. DMSO absorption was followed by either challenge in PTZ or a 1h absorption on *C. aurantium* (CA) extract 28mg/mL prior exposure to PTZ 3mg/mL. Statistical analysis revealed DMSO had no significant effect on seizure latency when compared both to control animals and the animals treated with UBP 301 alone. We administered DMSO and the *C. aurantium* extract to asses any possible interaction between these that could interfere with our experiments. There were son significant differences between the animals exposed to *C. aurantium* alone and those exposed to *C. aurantium* after DMSO nor between those exposed to DMSO + *C. aurantium* and UBP 301 + *C.*

aurantium. Results are shown as average ± SEM of at least three experiments, n>12. **** vs Naive $P < 0.0001$.
