# Supplementary material for: Citrus aurantium increases seizure latency to PTZ induced seizures in zebrafish thru NMDA and mGluR's I and II
Source: Front Pharmacol. 2015 Feb 13;5:284. doi: 10.3389/fphar.2014.00284 (PMC4327740; doi:10.3389/fphar.2014.00284)
Supplement: Supplementary file 2 [file Image2.PDF]

Supplementary Material

Modulation of PTZ induced seizures by *Citrus aurantium* in zebrafish: role of NMDA and metabotropic glutamate receptors.

Coral Rosa-Falero[1*], Stephanie Torres-Rodríguez[1], Rigel Licer[1], Yolimar Santiago[1], Zuleima Toledo[1], Marelys Santiago[1], Kiara Serrano[1], Claudia Jordán[1], Jeffrey. Sosa[2], and Jose G. Ortiz[1]

[1]Neuropharmacology Laboratory, Pharmacology and Toxicology Department, University of Puerto Rico-Medical Sciences Campus, San Juan, Puerto Rico
[2]RISE Program, Universidad del Este, Carolina, Puerto Rico

*** Correspondence:** Coral Rosa-Falero, [1]Neuropharmacology Laboratory, Pharmacology and Toxicology Department, University of Puerto Rico-Medical Sciences Campus, P.O. Box 365067, San Juan, 00936-5067, Puerto Rico.
coral.rosa.falero@gmail.com

1. Supplementary Data

1.1. Supplementary Figure 2

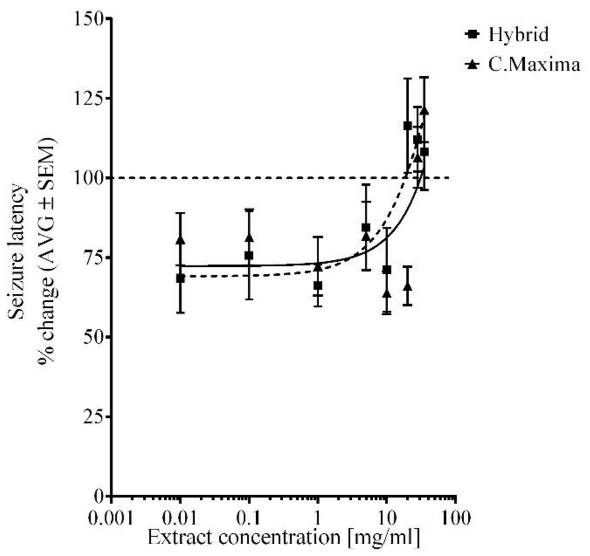

Supplementary Figure 2. Anticonvulsant properties of other members of the citrus genus. Additional members of the citrus genus were tested for anticonvulsant properties. Leaves from *C. maxima* tree and from a hybrid of *C. aurantium* and *C. sinsis* were collected, cleaned and the tea like leaf extract was prepared in the same way as the *C. aurantium* extract. Fish were allowed 1h absorption on the extracts followed by challenge in PTZ 3mg/mL. Neither the hybrid nor *C. maxima* caused a significant change on seizure latency although we can observe a marked tendency for concentrations

between 0.01mg/mL and 10mg/mL to reduce seizure latency when compared to untreated animals. Results are shown as average ± SEM of at least three experiments, n>12. * vs Naive $P < 0.05$; ** $P < 0.01$; ***$P < 0.001$.
