# Supplementary material for: Citrus aurantium increases seizure latency to PTZ induced seizures in zebrafish thru NMDA and mGluR's I and II
Source: Front Pharmacol. 2015 Feb 13;5:284. doi: 10.3389/fphar.2014.00284 (PMC4327740; doi:10.3389/fphar.2014.00284)
Supplement: Supplementary file 3 [file Image3.PDF]

Supplementary Material

Modulation of PTZ induced seizures by *Citrus aurantium* in zebrafish: role of NMDA and metabotropic glutamate receptors.

Coral Rosa-Falero[1*], Stephanie Torres-Rodríguez[1], Rigel Licer[1], Yolimar Santiago[1], Zuleima Toledo[1], Marelys Santiago[1], Kiara Serrano[1], Claudia Jordán[1], Jeffrey. Sosa[2], and Jose G. Ortiz[1]

[1]Neuropharmacology Laboratory, Pharmacology and Toxicology Department, University of Puerto Rico-Medical Sciences Campus, San Juan, Puerto Rico
[2]RISE Program, Universidad del Este, Carolina, Puerto Rico

*** Correspondence:** Coral Rosa-Falero, [1]Neuropharmacology Laboratory, Pharmacology and Toxicology Department, University of Puerto Rico-Medical Sciences Campus, P.O. Box 365067, San Juan, 00936-5067, Puerto Rico. coral.rosa.falero@gmail.com

1. Supplementary Data

1.1. Supplementary Figure 3

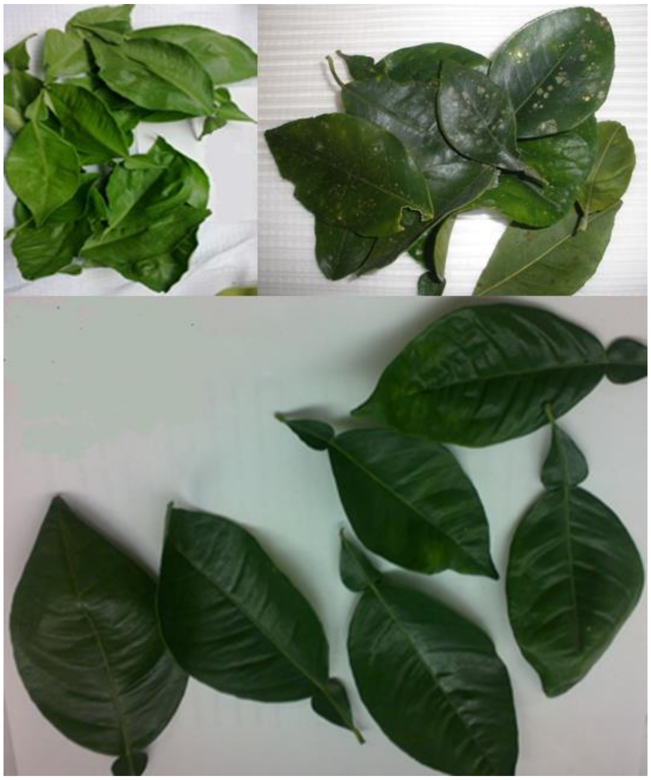

Supplementary Figure 3. Leaves from the *C. aurantium* tree used for experiments. Mature leaves, thick and firm, were collected from trees on the Eastern and Northern Region of the island of Puerto Rico. The picture is a sample of the leaves used for experiments.
