# Supplementary material for: Citrus aurantium increases seizure latency to PTZ induced seizures in zebrafish thru NMDA and mGluR's I and II
Source: Front Pharmacol. 2015 Feb 13;5:284. doi: 10.3389/fphar.2014.00284 (PMC4327740; doi:10.3389/fphar.2014.00284)
Supplement: Supplementary file 4 [file Image4.PDF]

Supplementary Material

Modulation of PTZ induced seizures by *Citrus aurantium* in zebrafish: role of NMDA and metabotropic glutamate receptors.

Coral Rosa-Falero[1*], Stephanie Torres-Rodríguez[1], Rigel Licer[1], Yolimar Santiago[1], Zuleima Toledo[1], Marelys Santiago[1], Kiara Serrano[1], Claudia Jordán[1], Jeffrey Sosa[2], and Jose G. Ortiz[1]

[1]Neuropharmacology Laboratory, Pharmacology and Toxicology Department, University of Puerto Rico-Medical Sciences Campus, San Juan, Puerto Rico
[2]RISE Program, Universidad del Este, Carolina, Puerto Rico

*** Correspondence:** Coral Rosa-Falero, [1]Neuropharmacology Laboratory, Pharmacology and Toxicology Department, University of Puerto Rico-Medical Sciences Campus, P.O. Box 365067, San Juan, 00936-5067, Puerto Rico.
coral.rosa.falero@gmail.com

1. Supplementary Data

1.1. Supplementary Figure 4

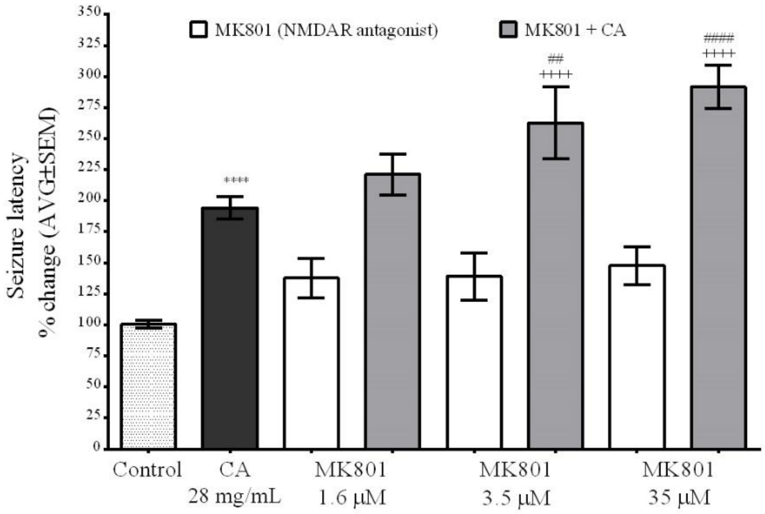

Supplementary Figure 4. NMDA receptor antagonist MK801 increased the anticonvulsant properties of the *C. aurantium* extract. Seizure latency of the *C. aurantium* extract was 194.3% ± 9. NMDA noncompetitive receptor antagonist MK801 was used at concentrations of 1.6 µM (seizure latency 137.7% ± 15.9), 3.5 µM (seizure latency 139% ± 19) and 35 µM (seizure latency 147.7% ± 15.3). Seizure latency after pre-exposure to MK801 followed with the *C. aurantium* extract was 221.3% ± 16.5, 262.8% ± 29, and 291.7% ± 17.5 respectively. This represented a significant increase of 35% and 50% in seizure latencies for the combinations with MK801 3.5 µM and 35 µM respectively. Results are shown as average ± SEM of at least three experiments, n>12. * vs Naive P < 0.05; ** P <

0.01; ***P < 0.001; ****P < 0.0001, # vs CA28mg/ml P < 0.05; ## P < 0.01; ###P < 0.001; ####P < 0.0001, + vs CA28mg/ml P < 0.05; ++ P < 0.01; +++P < 0.001; ++++P < 0.0001.
